# Pilot testing of the International Council of Cardiovascular Prevention and Rehabilitation Registry

**DOI:** 10.1093/intqhc/mzad050

**Published:** 2023-07-03

**Authors:** Sherry L Grace, Sana Elashie, Masoumeh Sadeghi, Theodoros Papasavvas, Farzana Hashmi, Gabriela de Melo Ghisi, Jorge Lara Vargas, Mohammed Al-Hashemi, Karam Turk-Adawi

**Affiliations:** School of Kinesiology and Health Science, Faculty of Health, York University, Toronto, ON M3J 1P3, Canada; KITE Research Institute & Peter Munk Cardiac Centre, University Health Network, University of Toronto, Toronto, ON M5S 1A1, Canada; Department of Public Health, College of Health Sciences, QU Health, Qatar University, Doha, Qatar; Cardiac Rehabilitation Research Center, Cardiovascular Research Institute, Isfahan University of Medical Sciences, Isfahan, Iran; Department of Cardiology, Heart Hospital, Hamad Medical Corporation, Doha, Qatar; Department of Rheumatology, Fatima Memorial Hospital & FMH College of Medicine and Dentistry, Lahore, Pakistan; School of Kinesiology and Health Science, Faculty of Health, York University, Toronto, ON M3J 1P3, Canada; Servicio de Rehabilitación Cardiaca, Departamento de Cardiocirugía, Centro Médico Nacional 20 de Noviembre, Ciudad de México 03104, México; Department of Cardiology, Heart Hospital, Hamad Medical Corporation, Doha, Qatar; Department of Public Health, College of Health Sciences, QU Health, Qatar University, Doha, Qatar

**Keywords:** cardiac rehabilitation, quality improvement, registry, feasibility study, pilot test

## Abstract

The International Council of Cardiovascular Prevention and Rehabilitation developed an International Cardiac Rehabilitation (CR) Registry (ICRR) to support CR programs in low-resource settings to optimize care provision and patient outcomes. This study assessed implementation of the ICRR, site data steward experience with on-boarding and data entry, and patient acceptability. Multimethod observational pilot involves (I) analysis of ICRR data from three centers (Iran, Pakistan, and Qatar) from inception to May 2022, (II) focus group with on-boarded site data stewards (also from Mexico and India), and (III) semistructured interviews with participating patients. Five hundred sixty-seven patients were entered. Based on volumes at each program, 85.6% of patients were entered in ICRR. 99.3% patients approached consented to participate. The average time to enter data at pre- and follow-up assessments by source was 6.8–12.6 min. Of 22 variables preprogram, completion was 89.5%. Among patients with any follow-up data, of four program-reported variables, completion was 99.0% in program completers and 51.5% in none; of 10 patient-reported variables, completion was 97.0% in program completers and 84.8% in none. The proportion of patients with any follow-up data was 84.8% in program completers, with 43.6% of noncompleters having any data entered other than completion status. Twelve data stewards participated in the focus group. Main themes were valuable on-boarding process, data entry, process of engaging patients, and benefits of participation. Thirteen patients were interviewed. Themes were good understanding of the registry, positive experience providing data, and value of lay summary and eagerness for annual assessment. Feasibility and data quality of ICRR were demonstrated.

## Introduction

Clinical registries are important tools in improving processes of care, particularly for conditions that are highly prevalent and where care is complex and variable [1]. Cardiac rehabilitation (CR) is a complex model of care for secondary prevention of the leading cause of death globally, namely, cardiovascular disease [2]. CR is composed of established core components including initial assessment, medical risk factor management, patient education, structured exercise, as well as lifestyle and psychosocial counseling, delivered by a multidisciplinary team [3]. Benefits are well-established in both high- and low-resource settings [4, 5], but there is variation in delivery between these settings [6].

There are few CR registries worldwide [7], with only one in a low-resource country [8]. While assessment and promotion of CR quality are warranted globally, there is much greater need in low-resource settings [9]. Therefore, in alignment with their mission [10], the International Council of Cardiovascular Prevention and Rehabilitation (ICCPR; https://globalcardiacrehab.com/) developed an International CR Registry (ICRR) to support these settings as they develop new programs to work toward consistency in care provision, hence optimizing patient outcomes [11, 12].

**Figure 1:**
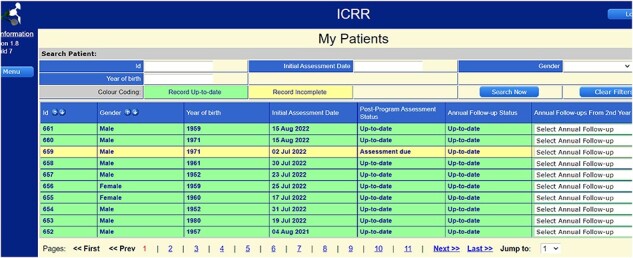
International CR Registry Interface to Prompt Follow-Up Assessment. Participating CR programs are prompted to complete follow-up assessments through color coding in the registry, which is based on their specific program duration and the date of initial assessment of a given patient entered. The data dictionary provides means to assess all but one follow-up variable via phone or online. These features minimize loss to follow-up.

While the value of registries has been established [13], previous research highlights the need for quality to ensure that the registry can fulfill its’ intended purpose [14, 15]. Other research has highlighted the challenges of implementing registries, including in the CR setting specifically [16], and the importance of ensuring their utility to end-users to support participation [17]. Therefore, it is important to field test implementation in these settings to identify issues. The objectives of this pilot test were to assess implementation of ICRR in the field with regard to (i) (a) patient eligibility appropriateness, (b) completeness/generalizability of patient volumes entered, (c) time to enter data, (d) variable completeness, (e) patient retention at follow-up, and (f) data accuracy; (II) data steward satisfaction with the (a) on-boarding and (b) data entry processes; and (III) patient acceptability of the ICRR.

## Methods

### Design

This is a multimethod observational field test of the registry over a 6-month period following launch, informed by the framework of Tan *et al*. [18]. Also, a focus group with on-boarded data stewards was held, and patients were interviewed to understand how to optimize end-user experience.

The ICRR has been developed and implemented in accordance with the Systems Development Lifecycle, in a linear and iterative manner [19, 20]. A description of the evidence-based planning, design, and developmental phases is reported elsewhere [11], as are results of usability tests [12], which preceded this field test.

### Setting: the ICRR

The ICRR is a health services registry of the ICCPR, which was soft-launched in late October 2021 with ICCPR Council (so a few programs could be on-boarded for piloting before broader launch). Program and patient inclusion criteria are outlined in the pre-registered protocol (ClinicalTrials.gov ID #NCT04676100).

ICRR comprises 12 program-reported variables (with an additional optional one where programs can enter additional information of their choosing) and 17 patient-reported variables (note: four program-reported and seven patient-reported variables are assessed at each assessment point). These are assessed preprogram, postprogram (dependent upon the duration of each program), and each year from initial assessment (not being tested in the current study) [11]. Programs are encouraged to collect data at all follow-up assessment points in patients who do not complete the program via phone (means to assess all variables but lipids provided in data dictionary) and enter it into the online interface (https://icrr.e-dendrite.com/).

The registry has a data quality dashboard that displays completeness of entry of each variable at each time point by site, as well as a screen showing when follow-up assessment is outstanding to promote retention (Fig. 1) [11]. Programs are provided information on this during the on-boarding process. ICRR has a data quality policy (https://globalcardiacrehab.com/ICRR-Governance); however, it was not initiated until after the pilot to enable this assessment.

### Procedure

The ICRR standard operating procedures for on-boarding were implemented (https://globalcardiacrehab.com/ICRR_sites). First, interested sites complete a program survey, which includes details on intentions for patient report, contact information of data steward(s), and patient volumes. Interested programs are then contacted with the site agreement and necessary documents to support application for research ethics waiver or approval.

The software host Dendrite initiates the site upon direction from ICRR Executive, which confirms receipt of these necessary approvals. Dendrite creates a CR site profile, with postprogram assessment timing triggered based on program duration as stated in the program survey, and sends data steward(s) a unique password-protected login. Named data steward(s) were then e-mailed ICRR training materials (e.g. screenshots of registry navigation, data dictionary, and instructions on using ancillary registry features), and a first on-boarding video meeting was scheduled with the ICRR user subcommittee cochair and all site data stewards, using the on-boarding meeting agenda template (Supplementary Data 1.A). Among other activities during the meeting, data steward(s) enter preprogram data for a patient anonymously.

Thereafter, preprogram assessment data were entered by CR staff on eligible patients. Then, a second and final on-boarding video meeting was held to coincide with initiation of postprogram/progress assessments (agenda in Supplementary Data 1.B). Each on-boarding meeting is ∼1.5 h in duration, and a link to the meeting recording is included in the minutes.

Data were entered prospectively from patients referred to participating programs during the 3-month period from November 2021 to February 2022, and follow-up ensued for 3 months given the participating programs’ duration (6–9 weeks to May 2022). In February, fully on-boarded sites were invited to the pilot study and all agreed. They were also asked to collect registry feasibility indicators in an Excel file, such as time to enter the data by assessment point. At the end of the pilot period, an independent member of the CR team was asked to compare entered values against patient charts for accuracy.

Pilot sites were also asked to interview willing patients to assess reasons why they participated in the registry and, if they did, their experience. Two of the three pilot sites were able to secure an ethics amendment to collect these data {Qatar and Iran [Isfahan University of Medical Sciences and Health Services (Protocol No: IR.MUI.REC.1400.046)]}. Patients who provided pre- or postprogram data were interviewed with a semistructured guide (Supplementary Data 1.C) in person or on the phone by program staff, with interpretation in the patient’s first language where needed.

Finally, in July 2022, a focus group was held with data stewards of all sites who had had both on-boarding meetings. The focus group was led by the first author (ICRR cochair) and the senior author (also ICRR cochair), and Sana Elashie took notes and recorded nonverbal communication.

The focus group was held via MS Teams for 1.5 h. To facilitate communication despite several different languages, live auto-transcription was enabled, all parties had their cameras on, and the focus group guide was both shared with participants in advance and on the screen during the focus group. Proceedings were video-recorded.

### Participants: ICRR programs, their data stewards, and patients

ICRR CR program inclusion criteria are a comprehensive program (i.e. initial assessment, structured exercise, and at least one other strategy to control risk factors) in a low-resource setting [21]. Each data entry steward of programs approved to join the registry who had had both on-boarding meetings was invited to the focus group. Data stewards who had difficulty in spoken English were excluded.

Consecutive patients referred to the participating CR programs during the period of study who consented to contribute data constituted the patient participants in terms of assessing the ICRR feasibility indicators. Patients participating in ICRR during the pilot who agreed to be interviewed were included. The ICRR includes exclusion criteria related to language, so no additional exclusion criteria were necessary.

### Measures

Registry feasibility indicators are shown in Table 1, the type of data quality they assess is also indicated. These include registry data generalizability, protocol inclusion/exclusion appropriateness to patients in low-resource CR programs, data completeness and accuracy, and time to enter and retention. To measure the former, monthly program volumes as reported in the ICRR program survey were used and compared to the number of patients entered in the registry per month. As outlined earlier, pilot sites were provided an MS Excel file with tabs to enter individual-level patient data (e.g. referrals received and which of these patients consented and were entered in the registry), time to enter the last 10 patient’s data, or data accuracy, for example, to support computation of some of the indicators. To compute other indicators, information from the registry itself was used (i.e. data completeness and retention).

**Table 1.** ICRR feasibility and data quality indicators.

Number	Indicator	Result
1^c^	Mean number of patients entered per month, as a proportion of monthly patient volumes	23.1 ± 6.1, 85.6%
2^d^	Proportion of referred patients who did not opt out or not consent meeting registry inclusion criteria	99.3%
3^e^	Proportion of referred patients not meeting registry inclusion/exclusion criteria who did not opt out or not consent entered into the registry^a^	1.3%
4^f^	Mean time (minutes) to enter program-reported variables preprogram (last 10 patients; Variables 1–5 and 8–12^b^)	9.0 ± 1.1
5^f^	Mean time (minutes) to enter patient-reported variables preprogram (last 10 patients who did not do self-report; Variables 13–24^b^)	9.1 ± 3.8
6^f^	Mean time (minutes) to enter program-reported variables postprogram, in those who completed the program (last 10 patients; Variables 6–12^b^)	6.8 ± 1.3
7^f^	Mean time (minutes) to enter patient-reported variables postprogram, in those who did not complete the program (last 10 patients who did not do self-report; Variables 13–26^b^)	12.6 ± 1.5
8^f^	Mean time (minutes) to enter patient-reported variables postprogram, in those who completed the program and did do not self-report (last 10 patients; Variables 13–26^b^)	10.0 ± 3.8
9^g^	Proportion of program-reported variables entered preprogram (Variables 1–5, 8–12^b^)	89.6%
10^g^	Proportion of patient-reported variables entered preprogram in patients who did not do self-report (Variables 13–24^b^)	89.5%
11^g^	Proportion of program-reported variables postprogram entered, in those who completed the program (Variables 6–12^b^)	99.0%
12^g^	Proportion of patient-reported variables postprogram entered in those who did not complete the program and did not do self-report (Variables 18–26^b^)	51.5%
13^g^	Proportion of patient-reported variables postprogram entered in those who completed the program and did not do self-report (Variables 13–26^b^)	97.0%
14^h^	Proportion of patients with any postprogram data (program or patient-reported; of Variables 8–26)	84.8%
15^h^	Proportion of noncompleting patients with any postprogram data (program or patient-reported) other than completion variables (of Variables 8–26)	43.6%
16^i^	Proportion of preprogram program-reported variables entered with correct values (Variables 1–5, 8–12^b^)^a^	97.9%
17^i^	Proportion of postprogram program-reported variables entered with correct values (Variables 6–12^b^)^a^	98.7%

a Lower values indicate better feasibility/quality.

b From data dictionary, available at https://globalcardiacrehab.com/ICRR-Variables-&-Data-Dictionary.

c Generalizability indicator.

d Patient consent rate/acceptability indicator.

e Inclusion/exclusion criteria appropriateness indicator.

f Time indicator.

g Data completeness indicator.

h Retention indicator.

i Data accuracy indicator.

The focus group guide for the data stewards (Supplementary Data 1.D) and semistructured interview guide for patients who provided any data (Supplementary Data 1.C) were developed by the ICRR cochairs; input was sought from the ICRR Steering Committee.

### Analyses

Descriptive statistics were used to characterize the feasibility and data quality indicators using Excel. Focus group and patient interview recording transcripts were cleaned to be verbatim and anonymized by S.A. A deductive-thematic approach was used for analysis of the focus groups using NVIVO 1.5.1 by the first author and S.A., as outlined by Crabtree and Miller [22–24]. Disagreements were reconciled with the senior author. Each theme and subtheme were supported by illustrative quotations (verbatim, except some minor edits were made to increase clarity in the case where the respondent’s first language was other than English). To ensure credibility, themes with subthemes were then shared with all data steward interviewees to inquire whether they resonated and requested any input (i.e. member checking) [25].

Finally, descriptive and content analysis of patient interviews was undertaken by S.A. and reviewed by the senior author [26]. Descriptive statistics (frequency and percentage) were used to summarize categorical responses. Moreover, the open-ended concepts and words were analyzed for tangible suggestions for improvement, and illustrative quotes were selected to exemplify responses.

## Results

At the time of the pilot, CR programs in Iran, Pakistan, and Qatar were on-boarded and agreed to take part. These programs were in tertiary care centers in major cities. The monthly patient volume at these programs was 25.6 ± 15.1.

### ICRR feasibility and data quality indicators

Indicators demonstrate high registry data quality and feasibility (Table 1).

The average time to enter data at preprogram and progress assessments by source was 6.8–12.6 min. Of 22 program- and patient-reported variables preprogram, completion was 89.5%. In terms of values, 98% were entered correctly.

With regard to patients, almost 100% met the registry inclusion criteria demonstrating applicability to low-resource settings (Table 1). Only <1% of patients declined to be part of the ICRR. Retention for postprogram data collection good in completers, but only about half in none. The proportion of patients with any progress data was 84.8% among completers, with 43.6% of noncompleters having any data entered other than completion status. Among patients with any progress data, of four program-reported variables, completion was 99.0% in program completers and 51.5% in none; of 10 patient-reported (which could be entered by program though interview), variable completion was 97.0% in program completers and 84.8% in none. Lipids pre- and postprogram (56.5% and 28.7%, respectively),and body mass index postprogram (69.3%) had lowest completion.

### Program perceptions of registry implementation

At the time of the focus group, CR programs in India and Mexico had also been fully on-boarded. Twelve data stewards from the five programs participated in the focus group (note: for four of the five centers, the primary data stewards did not enter data, but the program medical directors or managers who were involved in site approval, initiation, and training did), and their characteristics are shown in Table 2. Two data stewards were excluded for reasons of language proficiency. At the time of the pilot, given the first language of patients spoken at these centers, no program had been able to try the patient self-report, so that could not be assessed.

**Table 2.** Focus group participant characteristics.

ID	Sex	Profession/role
1	M	MD; Director of Rehabilitation Medicine
2	F	PT; CR
3	F	MD; Head of Preventive Cardiology and CR
4	F	PT; CR
5	F	PT; CR
6	F	Administrative secretary of the CR program
7	F	RN; CR
8	F	RN; CR
9	M	PhD; Program manager
10	M	MD; Sports medicine specialist, CR
11	M	MSc, PhD; Medical Director, CR
12	F	PT; CR

Abbreviations: F, female; M: male; MD, medical doctor; PT, physiotherapist; RN, registered nurse.

Themes are shown in Table 3, with corresponding illustrative quotes by subtheme. With regard to on-boarding, sites found no issues with the institutional approval process, but there were the usual time and administrative hurdles to securing ethical approval. Data stewards concurred about the utility of all data stewards meeting together for the on-boarding sessions, so they could agree on processes around who was entering which patients and how to arrange follow-up assessments.

**Table 3.** Emerging themes regarding ICRR on-boarding and initiation.

Theme	Subtheme	Illustrative quote (ID#)
On-boarding	Approval processes	“We went through certain challenges, but then now we have all the approvals. I guess that’s the way the system works.” (1) “We had no difficulty in getting ethics approval.” (10) “Besides the internal Ethics Committee approval, we also had to get governmental approvals.” (1)
	Nervous, but questions answered	“Actually, we were full of anxiety in the first meeting and we got relaxed after that…. The way you clarified all the queries was very helpful. All the meetings I have found like that. We have learned with time.” (3) “You support us a lot.” (11) “There was exchange of questions to and fro with you all as well. But ultimately, we have come around …and yeah, it’s been appreciated that we are part of the registry.” (1)
	All stewards working together for success	“We came up with a small (11) for us in order to be updated with each other …. I think it’s much easier now because we come up with a system.” (6) “At the first we just had some difficulty because I was the one who was entering the data for the first three months and then (32) and (4) were given access. But then when they were finally given access, they could only access the data that they would enter; They couldn’t see my data.” (7) “But it’s not one person’s responsibility to enter the data because [they did] the initial data entry.” (2) “In our center there were two people … entering the data.” (10)
Data entry	User-friendly, easy to navigate	“It’s quite user friendly; it’s quite comprehensive. It has all the information…whatever someone would want to explore about it.” (3) “I think they would see this was so user-friendly.” (11) “It’s quite easy for us.” (7) “I think it’s very easy, usable. … we’re very familiar with the database and we don’t find any kind of problems with this, and also its quick to do it.” (11) “Your database and the way that the registry is going on with this data form is very, very, very good for us.” (11)
	Variables—useful	“The education part… assessing how knowledgeable is that individual did help us to modify our program in terms of how we put educate the patient.” (2) “[We hadn’t measured] adherence to medication, the [social] support of the patient … [now measuring that] did help us to help patients get comfortable with their life.” (11)
	Variables—challenges	“The data entry … has become relatively easier for us. We have modified our hospital data entry system according to that.” (2) “Diabetes is very common in our region. So we are …gathering that data and mentioning it in the [optional variable].”(3) “We are entering the hemoglobin (HbA1c).” (2) “There’s an area of nutrition where we are now asking our patients how many servings .. the detail that you had mentioned in the data dictionary, it was not sufficient to understand.” (12) “The volume of physical activity not only in time, … but also in kilocalories.” (11)
Patient Engagement in the Registry	Introducing the registry	“I think that most of the patients they are very, very good, and the majority here would agree. And then they were sitting very well [for the questions], and they know all the information of the registry and how to participate in it.” (11) “Patients are not a very willing to accept all the policies….” (11) “I think just maybe in our cultural, it’s something new and that’s why we had a bit of a tough time in the start.” (12)
	Language barriers	“The problem was, uh, with the translation. That was because most of the people cannot use it.” (10) “I mean the lay summary that is being generated.. yeah, I would want it into different languages, as in options that I can click so that the patient can get that lay summary in the language as desired.” (2) “We printed and translated the lay summary for each person, and we handed it to them.” (10)
	Socially desirable responding	“What I have observed is in our region the people are very much dissatisfied.. Indicators show that quality of life is not [good]. But when we ask them, they say “no, we are very satisfied.” (3) “They don’t [report] the symptoms, that they are depressed. They say “no, we are very satisfied. We are happy.” (3) “I think it’s not a true representation of their clinical findings, but because it’s patient-reported data, so we have to [enter] what they are reporting.” (3)
	Ease of phone follow-up	“I was asking them would it be any problem if we would like contact them wherever they are, and they said that it would be OK.” (7) “I think patients who completed the program, when they complete the post-program survey or questionnaire [via phone], they are really happy to complete it.” (6) “…[those] who drop out, we call every patient and they are responding.” (3) “They do respond except in the case when there’s some error in the noting down the phone number or they have changed their contact details. Except those cases, everyone responds, and they give us the proper answer.” (3)
Benefits to Sites	Outcome dashboards	“I think there were some confusions because we don’t have much idea regarding how to use the dashboards probably.” (6) “Comparative tool like it would be more helpful in assessment if we can download the outcomes for certain durations.” (3) “… to improve our outcome, we do download it every month.” (3)
	Lay summary	“Actually, they said that it is really helpful because there are parts there that compare what they were before the program and like what the results were after the program. We actually have had great comments from [patients].” (7) “They could evaluate their health condition and they were very satisfied about that.” (10) “It really benefits the patient to know their progress, to have a documentation of their progress, in layman’s terms.” (7) “Like getting a little bit of evidence, and the lay summary is like a souvenir.” (7)
	CR quality community	“The quality meeting that would happen will help us understand what exactly to do, and going forward challenges will be addressed.” (2) “I’m looking forward to the quarterly quality control. You know, meetings that would happen for us to be able to understand our own data. So there are certain data points that we aren’t there, but there are certain data points where we are doing fairly well.” (2) “That’s, you know, putting us onto that track of pushing for certain improvements like patient education.” (3)
	Program certification opportunity	“…taking the initiative for certification through the registry, I think it would attract others to join, and I think it would have an impact.” (3) “…most of us are doing the ICCPR certification for [recognition] all over the world.” (12)
	Data export	“We have we been using it.” (2) “We have downloaded the pre and post data.” (7)

With regard to data entry, there was much discussion with regard to variables. Data stewards found the comprehensiveness of outcomes assessed to be useful, because it enabled them to identify areas where they could better serve their patients (e.g. knowledge gaps, social support assessment, and physical activity). They were glad that the optional variable was available, and sites were using that to enter blood glucose and graded exercise test parameters for instance. But they also noted some challenges. It did take some time to adjust at the beginning because some variables were assessed different from their usual practice (e.g. diet and quality of life). There was also the challenge with getting peak metabolic equivalents at postprogram as many patients did not wish to come back for a postprogram assessment and others had dropped out for clinical reasons or otherwise. The data dictionary promotes use of self-report Duke Activity Status Index where a functional capacity test cannot be performed, but MET values from that would not be comparable to a 6-minute walk test from preprogram for instance. Data stewards from one program decided to proactively collect the self-report survey in home-based model patients at intake and also to denote the type of functional capacity test in the optional variable to facilitate true assessment of functional status change over time. Finally, stewards noted that it was quite easy to collect all the variables except lipids and that the data completeness feature was helpful to them in ensuring this completeness.

With regard to their perceptions of the process of engaging patients in the ICRR, data stewards reported that with the first few patients, there were some challenges in explaining the registry to them, but they overcame them. For instance, many patients were not familiar with the concept of a registry, so they refined the way they introduced the registry to patients verbally, to ensure clarity about data privacy and security safeguards for example. While the overwhelming majority of patients agreed to be a part, some patients at privately funded centers did not want to answer questions over and above what would be required with the program’s standard intake assessment. Second, language barriers were paramount. While sites had translated the consent document to secure ethical approval, the ICRR website, patient-reported surveys online as well as lay summary are in English only, but sites were leveraging the Google Translate feature within the Chrome browser to overcome this. They noted how they interpreted the patient questions interview style. Third, they perceived that some patients may have been reporting overly high quality of life and low depressive symptoms compared to what they observed and knew about the patient’s context. Finally, they reported that patients were also very open to being called for follow-up assessments and indeed appreciated the continuity of care.

The final theme was related to benefits for participating sites. While the sites had not fully exploited the data export feature yet, the outcome dashboards were being used monthly by one program to inform their quality improvement activities, and other sites were looking forward to seeing any changes in their outcomes after 6 months. They reported being eager for the quality improvement policy of ICRR to be initiated, to be part of a CR quality community, and to meet regularly. They also planned to apply for optional program certification as part of their ICRR participation (https://globalcardiacrehab.com/Program-Certification). Finally, programs were using the lay summary as a record of graduation and to support continued self-management beyond graduation.

### Patient perceptions about registry participation and materials

During the period of study, data from 567 patients were entered into the registry by these three programs (note: some centers had closures due to major holidays or reduced volumes due to a coronavirus disease (COVID-19) wave). Their mean age was 58.5 ± 11.0 years (standard deviation), 82.4% were male, they had an average of 13.5 years of education, and 22.6% worked full- or part-time. Over half (55.3%) worried sometime or all the time about financial sufficiency to meet their basic needs, and 78.0% had to pay for their medications out-of-pocket.

Over half (57.5%) had acute coronary syndrome as the referral diagnosis, and many patients had percutaneous coronary intervention (47.7%) or bypass surgery (37.5%). Almost one-fifth (17.1%) were current tobacco users at program entry, and at least moderate-intensity physical activity was below recommended guidelines (123.3 ± 98.2 min/week). The mean systolic and diastolic blood pressure was 112.4/74.0 mmHg, and the average peak MET was 4.5 ± 3.2. The mean quality of life was 5.7 ± 1.2/10 (Cantril’s ladder [27]). These sociodemographic and clinical characteristics are consistent with what would be expected in CR, including in low-resource settings [4].

One pilot site interviewed three patients and the other 10 (12 males and 1 female). Overall, patients had positive perceptions and a good understanding regarding the registry (Table 4). The interviewees concurred with data stewards that the questions and patient lay summary were useful; patients stated that this helped them manage health. The future annual follow-up would be well-received and valued by patients, and phone call was the common method of contact desired by the patients. The patient webpage was perceived as clear, and some of their web browsers translated it for them.

## Discussion

### Statement of principal findings

In this first test of the implementation of the ICRR—and indeed, it was a true field test given the impact of the COVID-19 pandemic on CR delivery during the period of study [28]—the feasibility of participation of target CR programs and their patients (i.e. in low-resource settings) [21] as well as quality of resulting data was supported. The overall time to secure approvals (ranges based on whether ethics waiver can be secured and given local ethics board review response times) as well as for on-boarding (1–2 h to read ICRR materials and two 1.5-hour on-boarding meetings) was considered significant, but worthwhile by participating programs, given the many benefits of ultimate participation. Time to enter data was considered acceptable. Patients were also highly willing to participate, did not have issues with the number or nature of the questions, and desired the feedback on their progress facilitated through their registry participation.

### Interpretation within the context of the wider literature

Results of this ICRR implementation evaluation are in line with other work evaluating the quality of more mature registries [18, 29]. Moreover, input from data stewards in the focus group was consistent with that of qualitative studies of stewards in other CR registries [16, 17]. Based on the results of the focus group, several improvements were made to ICRR and its associated processes. First, at site set-up, we specify that all site data stewards can see all patient records for the given site. Second, the agendas for both on-boarding meetings were strengthened (Supplementary Data 1.A and 1.B) to ensure that all ICRR features are fully demonstrated to the sites. While we will continue to do two on-boarding meetings with each site individually and provide minutes with video-recording link, given language differences, we created a generic instructional recording for each of the two on-boarding meetings, which are posted to the site page of ICRR (https://globalcardiacrehab.com/ICRR-Training) for programs to view as needed.

Third, edits were made to the data dictionary to improve clarity. Elaboration around medication adherence was clarified, suggestions around circumventing socially desirable responding for some items were added (e.g. social support), chewing tobacco was added as an example of a form of tobacco, and an explicit notation was added around operationalizing “at least moderate-intensity exercise” with patients, potentially using the terminology of “slightly short of breath.”

**Table 4.** Patient perceptions regarding registry, *N* = 13.

Question	Response summary	Perceptions, illustrative quotes (ID#)
Understanding of registry	13 (100.0%) remembered about ICRR 13 (100.0%) were able to express understanding	“Program is done worldwide for all programs to help each other improve their services.” (2) “To monitor us at the same time” (3) “Our information is recorded anonymously before and after rehabilitation in an international registry.” (193)
Process of learning about the registry	9 (90%) perceived registry consent form/information sheet was clear	“The information was sufficient and clear.” (176) “The information was clear and I fully understood.” (308) “Explanation given to me was clear and complete.” (193) “The secretary of the department explained very patiently, accurately and completely.” (203)
Experience providing data	11 (84.6%) perceived that the questions were clear and understandable 10 (76.9%) were satisfied with the amount of time to answer them	“I was not worried about participating … because the questions were normal.” (193) “…English words that are not as simple to be understood by a layman.” (2) “It was quite understandable because it actually consisted of questions about my daily life such as exercise, eating fruits and vegetables, and so on.” (386) “The time to answer the questions was good, I was not tired, and I was even willing to spend more time.” (192) “The time to answer the questions was enough and I was not bored at all” (193) “The number of questions was low and the time to answer was short; it would have been better if they had asked us for more explanations.” (203) “It was good and if I did not understand a question, the Secretary would explain it to me and then I would understand.” (176)
Lay summary	11 (84.6%) desired it 11 (84.6%) understood implications	“At least with a reminder … it will help us to be mindful of our actions.” (2) “I was interested to know if my condition had improved during this program.” (176) “It was according to what I imagined about myself.” (308) ”It was really interesting because I knew how much I was improving in the rehabilitation period.” (386) “Its content is complete and good, especially the parts related to diet and quality of life.” (207) “If you can create an application because if it is only on paper or even soft copy, we can easily discard that. If it is an app, we can set reminders.” (239) “I think this was a usual thing and I knew the information in this summary, and it was not interesting to me, and it did not add much to my information.” (3) “I am illiterate. My son read this to me and that was good and complete.” (308) “It would be better to provide information about the amount of physical activity we are allowed to do, as well as about sexual activity, smoking, and alcohol consumption. Also, about the effect of genetics on cardiovascular disease.” (203) “I suggest that this summary also include chest pain, post-CABG care, permissible daily activities, and travel in heart patients.” (192)
Annual assessments	12 (92.3%) would be willing to answer questions on the phone	“It is good that I get follow-up so that I can know if I am really okay and if I am doing things right even after a year.” (3) “It is good to update patients on their status.” (3) “It is our second life; we have to take care of it and value it more.” (2) “Definitely with pleasure.” (176) “I think one year is a long time and it would be better to do this follow-up sooner.” (192) *MODE:* “Email or call would be okay.” (3) “It is better to be by phone.” (203) “Phone and in person.” (308) “Phone or WhatsApp call.” (192)
Patient page on registry website^a^	4 (30.8%) visited the patient webpage for the registry	“I got good information about the registry.” (386) “Patient’s website was easy to understand.” (239)

a https://globalcardiacrehab.com/ICRR-for-Patients.

### Implications for policy, practice, and research

The implications of this research are several. In terms of the ICRR specifically, where funding permits, we would make some improvements to, and translate, the lay summary to the most common first languages of registry-interested programs, although in the interim programs are exploiting translation software embedded in browsers. We would create a compatible discharge summary for referring physicians as well [30]. We would augment outcome dashboard functionality by adding more time increments for comparison (currently 6 months). We will need to continue to support programs to minimize loss to follow-up, particularly in patients who do not complete the program, to support the utility of the ICRR. Indeed, validity of the lipid data, in particular, will be limited due to impracticality of collecting these data in these settings where patients must travel and often pay out-of-pocket for the tests as well.

ICRR continues to mature. As of June 2023, we now have 17 participating sites covering all regions of the globe and ∼2350 patients; ∼12 sites are currently seeking approvals. CR is available in 55/138 low- and middle-income coutries, and we hope to achieve representativeness [6]. We have initiated our data quality checks, so the data completeness and retention issues identified through this study will be rectified in a timely fashion in the future. In addition, to support implementation of the biannual data quality audit, communication to sites has been drafted by the research subcommittee and approved by the Steering committee and is now being tested. Moreover, based on the results of this study, we held a training session on ICRR features that support program quality improvement as well as training for the annual assessment (https://globalcardiacrehab.com/ICRR_sites). We have also launched our program certification initiative and are pilot testing it (https://globalcardiacrehab.com/Program-Certification).

## Strengths and limitations

Caution is warranted in interpreting these results. While representative generalizability is not established through qualitative methods, results applicability to all low-resource settings to which the registry is targeted cannot be known—also considering the small number of countries represented in this pilot study. Second, efforts were made prior to interviews and throughout the pilot period of study to minimize the potential of socially desirable responding, but this may have skewed results to be more positive. Efforts were also made to ensure interviewer neutrality, and coding for both the focus group and interview included a non-ICRR chair. Third, there was only one focus group given the limited number of eligible participants, and thus, it cannot be concluded whether full saturation was achieved. Nevertheless, novel preliminary information to inform future practices was identified through these aspects of this study. Finally, the nature of the design precludes causal conclusions.

## Conclusion

In conclusion, the feasibility, quality, and acceptability of the ICRR have been supported. Nevertheless, ICRR will seek to translate registry materials and promote retention of patients who do not complete their CR program, which unfortunately are many in low-resource settings due to paying out-of-pocket for CR, among many other barriers. This will facilitate achievement of ICRR’s mission in supporting CR in low-resource settings with regard to care, research, and advocacy.

## Supplementary Material

mzad050_Supp

## Data Availability

For the qualitative study, participants of this study were not asked for permission to share their data publicly, so supporting data cannot be made publicly available. Data are available upon request to the corresponding author by qualified investigators with appropriate approvals.
