# Supplementary material for: Pilot testing of the International Council of Cardiovascular Prevention and Rehabilitation Registry
Source: Int J Qual Health Care. 2023 Jul 3;35(3):mzad050. doi: 10.1093/intqhc/mzad050 (PMC10329404; doi:10.1093/intqhc/mzad050)
Supplement: mzad050_Supp [file mzad050_supp.zip › suppl_data/ICRR Pilot_manuscript_Suppl1_vIJQHC_SGinput-clean.docx]

**Supplement 1**

**Supplementary File 1.A**


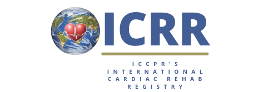


International Cardiac Rehab Registry (ICRR)

On-Boarding Meeting 1 of 2 (Pre-Program Assessment)

DATE & TIME (include time zones for all invitees): xxxxxxxxxx

INVITEES: xxx (ICRR user sub-committee co-chair for on-boarding), xxxxx (ICRR secretary), xxxxxxxxx (data stewards)

MTG LINK: xxxx

SUPPORTING DOCUMENTS: data dictionary, protocol, excel to keep track of patient registry ID#s, software training manual

Agenda:

1. Welcome & introductions (meeting chair)
   1. all site data stewards present?
2. Confirmations to proceed with on-boarding
   1. Any questions from chair regarding program survey responses?
   2. All data stewards have access to ICRR (logins) and looked around?
   3. Any questions regarding protocol (inclusion/ exclusion criteria etc.)?
   4. Any questions regarding patient opt-out or consent process (as applicable for site)
      1. Translated materials?
3. Questions re: variables and their definitions
4. Any variables cannot assess?
5. Any variables where definition is a challenge?
6. Review / discuss site processes in terms of collecting patient-reported data
   1. Discuss process of getting patient-reported data from patients to enter, especially if non-English speakers
      1. Show survey feature in case they can use in future (see patient source variable at bottom of p. 1 of registry and open the survey link: <https://rs2.e-dendrite.com/csp/icrr/PROMS/QuestionnaireIntake.csp?zkey=MTI1IDEyOCAzOTUyMzkwNjIxMTU0OTMwNSAx>)
      2. Google Translate in Chrome? Interpret interview style?
      3. we can provide the surveys (electronic word files) if you want to use them for each assessment point
         1. translate from English?
            1. print out?
7. Demonstrate registry navigation & data entry
   1. particularly pre-program assessment data entry (pages 1, 2 and 3, 4 [latter 2 are the pt-reported])
      1. observe steward enter data for first patient (share screen but keep patient identity private)
         - source of pt data: program?
         - answer questions (program and patient report)
8. Tracking patients for entry of follow-up data
   - 1. emailed the excel template to match patient name and registry ID
        - need: due to anonymity, won’t be able to easily find patient later
        - confirm password-protected and saved securely on institutional server only
9. Any Other Business, questions
10. Next steps
    1. Arrange 2^nd^ of 2 on-boarding web calls at time when they will be entering first post-program data, for support through process
       1. Registry set up that on the “my patients” screen it will show as yellow/”assessment due” once your program duration time provided to Dendrite based on your program survey responses has elapsed from the initial assessment date entered for each patient
    2. Enter data on your new patients going forward, and email us if you have any questions or challenges
11. Close

**Supplementary File 1.B**


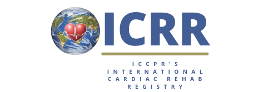


International Cardiac Rehab Registry (ICRR)

Site On-Boarding Meeting #2 of 2 (Progress Assessment & Registry Features)

DATE & TIME (include time for all invitees): xxx

INVITEES: xxx (ICRR user sub-committee co-chair for on-boarding), xxx (ICRR secretary), xxxxx (all site data stewards)

MTG LINK: xxxx

SUPPORTING DOCUMENTS: Ancillary features file

Agenda:

1. Review of agenda
2. Review any challenges thus far
   1. Data definitions
   2. Check data/variable completeness (ancillary feature in registry – see file)
      1. % ok in first 2 tables? (pre-program variables)
         1. If red, discuss issues
      2. When enter post-program data, monitor 2^nd^ two tables
         1. The research sub-committee monitors too, and may be in touch to see if they can help if % low / variables are red
            1. E.g, be sure to enter premature program termination for patients that drop-out
   3. Process with patients
3. Observe steward enter post-program/progress data for first patient (pages 5, 6 and 7,8 [latter 2 are the pt-reported])
   1. Tracking patients for entry of follow-up data – get out the excel
   2. answer questions (program and patient report)
   3. Show patient lay summary (on p. 5)
      1. Can send us your logo and it can be added in at top
      2. You could use DeepL or Google translate to translate to patient’s first language
      3. Could send it to referring physician; if you do, remember to put the patient’s name though (because the registry is anonymous)
4. Using other features of registry
   1. Export my data
      1. pre and post in separate files, so contact us if need help
   2. Dashboards, pt-related outcomes report
      1. Each of 12 figures – details in “ancillary features” file
      2. See also training video explaining at: https://globalcardiacrehab.com/ICRR-Training
   3. All registry main documents can be found on “main menu” in blue “documents” (if we update them, we post them there)
5. Any Other Business, questions
6. Next steps
7. You will receive an email to support you in quality improvement, including information about optional program certification (<https://globalcardiacrehab.com/Program-Certification>)
   1. Quality improvement co-chair may be in touch, or you can reach out to us
8. Annual assessments (page 9,10 and 11 of registry)– a training webinar available at: <https://globalcardiacrehab.com/ICRR-Training>
   1. We will also send a detailed email at one year
9. Minutes to follow
10. Be in touch with any questions at iccpr.icrr@gmail.com
11. Close

**Supplementary File 1.C**

**
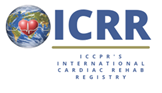
**

**ICRR Pilot**

Semi-structured interview questions for patients who provided data

We are grateful your data could be part of the registry, so we can learn more about our care delivery and how we can improve it.

1. First, tell us what you understand about the cardiac rehab registry in which our program is participating, if anything?
   1. (if they aren’t sure, tell them it is ok if they do not remember about it and describe to see if they remember)
2. Do you remember first being informed about the registry by program staff?
   1. IF YES only:
      1. Was it through an information sheet [if yes, mailed or given in person?], verbally, or both?
         1. Was the information about the registry clear? How could that have been improved?
      2. What were your initial thoughts about your data being part of the registry?
         1. PROBE: positive thoughts, any concerns
      3. What questions did you have about the registry and participating in it, that you were not informed about?
3. Do you remember answering the registry questions when you started the program?
   - 1. IF YES:
        1. How did it go for you?
        2. Did you just tell the staff your answers, or did you fill out a form?
           1. If you could get an email or text with a survey, would that work for you?
        3. Were the questions clear? Why or why not / in what ways?
        4. How understandable were the questions?
        5. What did you think about the amount of time it took to answer the questions about your quality of life and heart-health behaviors, etc.?
4. Some programs that are participating in the registry will be giving patients a summary of how they are doing at the end of their cardiac rehab program, with a goal to support patients like you to continue maintaining their heart health. Here is what it looks like
   1. Is this something you would be interested in receiving? Why or why not?
   2. What do you think about the content of the lay summary? In your opinion, how could it be improved?
   3. Based on reading this example lay summary, what do you think this patient should do to maintain their heart-health now?
   4. How do you think the summary could be improved, so if you received one, you would be eager and able to continue with your heart-health behavior you learned in cardiac rehab?
5. As a participant in the registry, you will be contacted every year to find out how you are doing. While this is optional, do you think you will be willing to answer the questions over the phone? why or why not?
   1. What way would you prefer to be contacted to answer the questions each year?
6. As a participant in the registry, would you be interested in hearing from the registry about what they are learning from the initiative, and how they are working to improve cardiac rehab? Why or why not?
7. Did you go to the patient webpage for the registry (https://globalcardiacrehab.com/ICRR-for-Patients)?
   - 1. If no, show them
     2. What do you think about the information here?
        1. PROBES: Layout, legibility, clarity, volume of information, missing content, content you think is not needed?
8. Thanks for your time. Is there anything else you would like to tell us about your experience learning about and being a part of the registry?

**Supplementary File 1.D**

**ICCPR ICRR Pilot**

Semi-structured focus group guide for data stewards

1. Describe your experience learning about the registry, and getting questions answered (if any at that time) in terms of applying to be a part?
   1. Describe your experience completing the program survey on REDCap
      1. Clarity, length, tech issues,
      2. missing or extraneous questions?
   2. What was frustrating? What could we have done better?
   3. Any ideas on how we can better reach and inform cardiac rehab sites about the registry and being a part?
2. How do you think we could improve the registry website, to better support interested sites?
   1. And participating sites?
3. Describe your experience securing institutional and ethics approval for participating in the ICRR at your centre. In what ways could ICRR have been more supportive?
   1. What questions did your institution or ethics board have about your program being part of the registry?
   2. Did you have to translate materials? If yes, how did you arrange that and how could it have gone more smoothly?
4. Describe your experience preparing for the first on-boarding meeting, including reviewing the data dictionary, and navigating the registry website for the first time.
   1. What was frustrating? What could we have done better?
5. Describe your experience with the first on-boarding meeting where you were introduced to the registry processes and pre-program data entry
   1. What was frustrating? What could we have done better?
6. Describe your experience informing patients about the registry
   1. How did you do it there, and did that change over time?
   2. Was it particularly time-consuming? Describe
7. Describe your experience with the second on-boarding meeting where you were introduced to post-program data entry and other registry features such as the dashboards
   1. What was frustrating? What could we have done better?
8. Once you got comfortable entering registry data, describe your experience entering it
   1. PROBES: time required, pulling together needed information
   2. Areas with insufficient clarity in data dictionary?
      1. PROBES: did they consult it often? Did they use the open-ended free-text variable?
   3. identifying patients, knowing when next assessment due
   4. did the registry feel easily “usable”? in what ways?
   5. data quality problems as going: completeness, accuracy, attrition
   6. how many staff are entering the data, and do you think you are doing it consistently?
      1. How is it going communicating who is responsible to enter what?
   7. How often do you go in to the registry website?
   8. How do you alert yourself when it is time to enter post-program data for a patient, or do you not have any reminder system but the color coding in the registry?
9. Did you use any of the ancillary registry features yet? And if yes, were they useful and satisfactory?
   1. Dashboards – how used?
      1. Pt-related outcomes report?
      2. Shared with anyone internal/external to program or will you?
   2. Lay summary – used in any way?
   3. Have you downloaded and used your data?
   4. Any features not really useful? Any features you would like?
10. Describe your experience getting post-program data from patients
    1. Patients that completed the program
    2. Patients that did not complete the program
    3. Patients doing a home-based model (if applicable)
    4. Experience with patients who did not want to provide follow-up data
11. What are your concerns about the annual assessments?
    1. How can ICRR support you in getting that data from patients?
12. Has being part of the registry felt like it is worth the effort so far?
    1. How long do you think it will be useful for you to keep entering new patients?
13. Anything else you would like to share with us?
